# Damping Analysis of Some Inorganic Particles on Poly(butyl-methacrylate)

**DOI:** 10.3390/ma11060992

**Published:** 2018-06-12

**Authors:** Saisai Zhou, Chunhua Yang, Jia Hu, Xianru He, Rui Zhang

**Affiliations:** 1 School of Materials Science and Engineering, Southwest Petroleum University, Chengdu 610500, China; 201621000041@stu.swpu.edu.cn (S.Z.); 201721000028@stu.swpu.edu.cn (C.Y.); 201531053301@stu.swpu.edu.cn (J.H.); 2 Institute für Physik, Universitӓt Rostock, Albert-Einstein-Str. 23-24, 18051 Rostock, Germany

**Keywords:** poly(butyl-methacrylate), inorganic particles, damping property, glass transition temperature

## Abstract

Viscoelastic polymers can be used as damping materials to control unexpected vibration and noise through energy dissipation. To investigate the effect of an inorganic filler on damping property, a series of inorganic particles, Ferriferous oxide(Fe_3_O_4_), Graphene/Fe_3_O_4_(GF), and Fe_3_O_4_ of demagnetization(α-Fe_2_O_3_) were incorporated into poly(butyl-methacrylate) (PBMA). The effects of the dispersion of particles, as well as the interaction between particles and the PBMA matrix on the damping property of composites, were systematically studied. Results revealed that the addition of three types of particles can effectively improve the damping properties and broaden the effective damping temperature range. Dispersion of α-Fe_2_O_3_ in the PBMA matrix is better than that of Fe_3_O_4_. As a result, the damping peak can be increased more. The interaction between GF and the PBMA matrix is stronger than that between Fe_3_O_4_ and the PBMA. The damping peak of the composites can be suppressed by GF, which is opposite to Fe_3_O_4_ and α-Fe_2_O_3_. In addition, glass transition temperature (T_g_) of all composites in the study shifted to low temperatures.

## 1. Introduction

With the development of modern technology, all kinds of mechanical equipment are developing high speed, high efficiency, and automation, but vibration and noise, which are produced during work, seriously damage the reliability and stability of the machine itself [1,2]. Passive or active damping are extremely effective ways for, mostly, vibration control [3,4]. Passive vibration control involves the modification of the stiffness and damping of a vibrating system. A straightforward and effective solution for vibration and noise control is the application of a viscoelastic material, such as polymers [5]. Because of its high damping characteristics around the glass temperature, viscoelastic polymer is usually used as a damping material to control noise and vibration, which has attracted considerable attention [6]. The loss tangent (tan δ), which is the ratio of E″ to E′ (tan δ = E″/E′), is used as an assessment of the ability to dissipate energy by elastomers. E′ and E″ represent the storage modulus and loss modulus, respectively [7]. High-performance viscoelastic damping materials should meet the requirement of tan δ > 0.3 over a broad damping temperature range of at least 60–80 °C [8]. 

Matrix polymers must be considered according to the application and environment in which the material is to be applied [9]. Polyacrylate materials are well known for their excellent damping properties for plenty of polar ester groups [10,11] and PBMA has an excellent adhesion property, with its macromolecular chain having many branched chains, which can effectively increase internal friction and improve damping properties. A great efforts have been devoted to broaden the effective damping temperature range of acrylic polymers, including co-polymers, interpenetrating polymer networks (IPN), and blends [12,13,14]. One of the most important ways is blends consisting of adding filler with modifications.

Fe_3_O_4_ (ferriferrous oxide), in our previous study, was found to have a strong interaction with ethylene vinyl acetate (EVA), which has polar vinyl acetate (VA) segments [15,16]. Fe_3_O_4_, theoretically, should also have strong interactions with PBMA because of its polar carbanyl groups. So, after hybridization with Fe_3_O_4_, the particles will have interactions with PBMA, which can make a great contribution to mechanical loss [17]. However, it is easy for Fe_3_O_4_ particles to aggregate due to magnetism between particles [18,19]. Thus, the dispersion state of particles in the matrix and the interactions between particles and the matrix are very important for damping properties of composites. To improve the dispersion of Fe_3_O_4_ in the PBMA matrix, Fe_3_O_4_ particles of demagnetization were prepared in high temperatures [20,21]. Moreover, to study the effect of fillers-matrixes interactions on the damping properties of PBMA, Fe_3_O_4_ and graphene/Fe_3_O_4_ hybrid particles were incorporated into PBMA. These two particles have a distinguishable interaction with PBMA, since they have different interfaces. Graphene, with a two-dimensional layer, prevents Fe_3_O_4,_ with three-dimensional sphere shape, from aggregating. Meanwhile, due to the magnetism of graphene [22], it is easier for Fe_3_O_4_ to load graphene as the core.

In this article, to study the effects of the dispersion of particles, as well as the interactions between particles and the PBMA matrix on damping property of composites, Fe_3_O_4_, Graphene/Fe_3_O_4_ (GF), and α-Fe_2_O_3_ are incorporated into PBMA. The elemental analysis, morphology, and specific surface area of graphene hybrid particles are analyzed by energy dispersive spectrometer (EDS), scanning electron microscopy (SEM), and specific surface area and pore size tester, respectively. The structures, particle size, and magnetization of Fe_3_O_4_ of demagnetization(α-Fe_2_O_3_) are characterized by X-ray diffraction (XRD), laser particle size analyzer, and vibrating sample magnetometer analysis (VSM). The dynamic mechanical analysis (DMA) and scanning electron microscopy (SEM) are used to characterize the damping materials. It is expected to find far-ranging applications as damping materials in vibration control.

## 2. Materials and Methods

PBMA (M_w_ = 504631, PDI = 8.186) was synthesized by free radical emulsion polymerization in our laboratory. Fe_3_O_4_ particles were synthesized by the procedure reported previously in Ref. [15]. Pristine graphene (number of layers, 1–10) was provided by Deyang Carbonene Co., Ltd (Deyang, China). Fe_3_O_4_ of demagnetization(α-Fe_2_O_3_) were obtained by heating Fe_3_O_4_ particles up to 500 °C (under air atmosphere). Sodium dodecyl benzene sulfonate, sodium persulfate, ferric trichloride, Ferrous sulfate, sodium hydroxide, and polyvinyl alcohol were all purchased from Chengdu Kelong Chemical Reagent Factory (Chengdu, China). The reagents were analytical grade.

### 2.1. Synthesis of Graphene/Fe_3_O_4_ Hybrid Particles

First, 0.2 g of pristine graphene was added into a NaOH (sodium hydroxide) aqueous solution (0.1 mol/L) and heated to 80 °C with vigorous stirring. Then FeCl_3_·6H_2_O and FeSO_4_·7H_2_O were dissolved in a PVA aqueous solution to prepare the mixed solution of iron ions with 2:1 molar ratio of Fe^3+^ to Fe^2+^, then 200 mL mixed solution of iron ions was dropped slowly into the NaOH aqueous solution with graphene through a constant pressure drop funnel at 80 °C in a water bath. The reaction continued for 40 min and the black precipitates were washed with alcohol and deionized water for at least five times, followed by freeze drying at 50 °C for 96 h.

### 2.2. Preparation of Samples of Poly(butyl-methacrylate)-Based Hybrid Material

Blends of PBMA with different particle contents were prepared via solution mixing at room temperature for 15 min. The basic formulation of the composites is listed in Table 1; the blends were compression molded to form sheets (20 mm long, 12 mm wide, and 3 mm thick) under a pressure of 10 MPa for 20 min at 160 °C.

### 2.3. Characterization

X-ray diffraction (XRD, X Pert PRO MPD, Almelo, the Netherlands) was used to test the crystal structure of Fe_3_O_4_. The scanning range was from 5° to 70° and the scanning speed was 3.6°/min.

Particle sizes were characterized by Laser Particle Size Analyzer (Master sizer 2000, Malvern Instruments Ltd., Malvern, UK), ethanol was used as a dispersant, and the shade was 3.5%.

The specific surface areas were tested by a specific surface area and pore size tester (ST-MP-9, Quantachrome Instruments, Beijing, China). The test results were analyzed by the multipoint Brunauer-Emmet-Teller (BET) method. Before the test, samples were kept at 423 K in a vacuum environment for about 2 h.

VSM (BKT-4500Z, Quantum Design Ltd., San Diego, CA, USA) was carried out to measure the saturated magnetic strengths of particles, in open circuit mode at normal temperature.

The morphology of the poly(butyl-methacrylate)-based composite material was studied by scanning electron microscopy (SEM; ZEISS EV0 MA15, Carl Zeiss microscopy Co., Ltd., Jena, Germany). To prepare samples for SEM analysis, the composites were quenched in liquid nitrogen and cryogenically fractured.

Elemental analysis was carried out at Energy Dispersive Spectrometer (EDS; Elementar, Langenselbold, Germany) for determination of the Fe, O and C content.

Dynamic mechanical analysis (DMA) was carried out on Q800 (TA Instruments, New Castle, DE, USA) by using a dual cantilever clamp and a testing method of temperature ramp-frequency sweep with a frequency of 1 Hz. The samples were trimmed to dimensions of 20 mm long, 12 mm wide and 3 mm thick. The oscillation strain amplitude was set to be 15 mm.

## 3. Results and Discussion

### 3.1. Morphologies and EDS of Graphene/Fe_3_O_4_ Hybrid Particles

**Figure 1:**
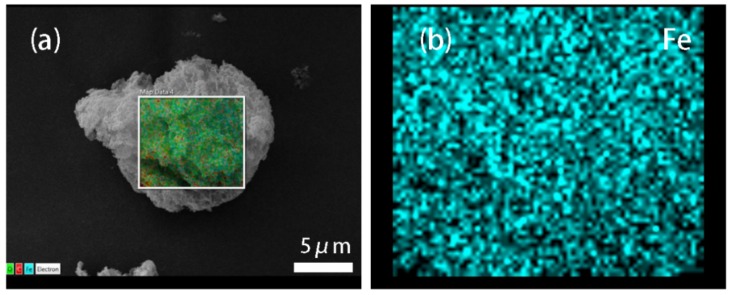

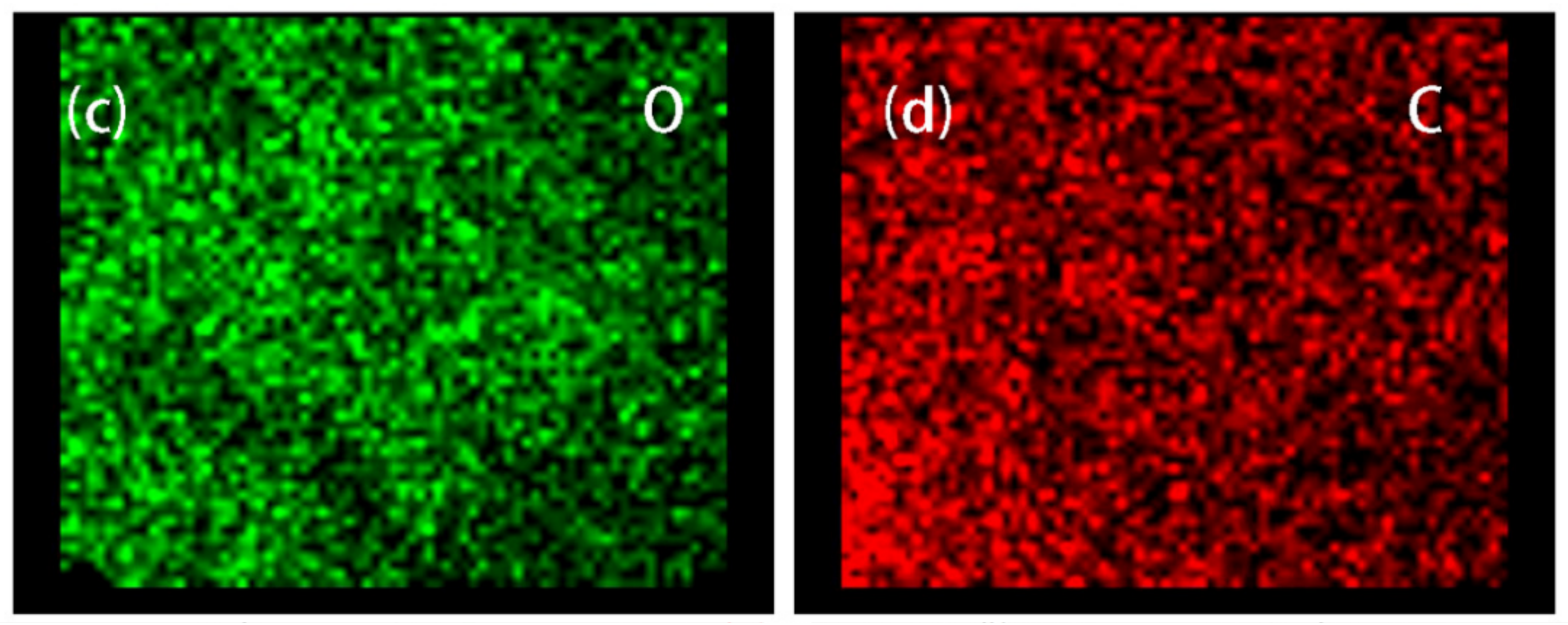
(**a**) SEM images of Graphene /Fe_3_O_4_; (**b**–**d**) iron, oxygen, and carbon mapping images of Graphene/Fe_3_O_4_.

The morphology of the GF composite particle is shown in Figure 1a. As we can see, GF presents a near-spherical shape. Fe_3_O_4_ particles, as the core and most of the particle surface, are surrounded by graphene sheets. To further testify the structure of GF, the GF particle was analyzed by energy dispersive spectrometer. Figure 1b–d are the mapping images of iron, oxygen, and carbon elements, respectively. The above discussion indicates that Graphene and Fe_3_O_4_ particle was compounded when available.

### 3.2. Demagnetization of Fe_3_O_4_

**Figure 2:**
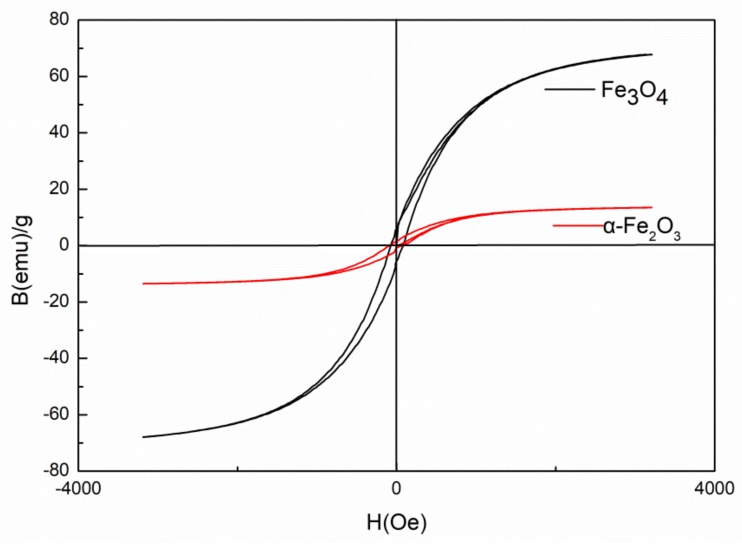
Magnetic hysteresis loops of Fe_3_O_4_ and α-Fe_2_O_3_.

As reported in the literature, a high temperature is commonly used for demagnetization [21]. Demagnetization of Fe_3_O_4_ (α-Fe_2_O_3_) was obtained by heating Fe_3_O_4_ particles up to 500 °C (under air atmosphere) to improve the dispersion of Fe_3_O_4_ in the PBMA matrix. The magnetic properties of Fe_3_O_4_ and α-Fe_2_O_3_ were studied by a vibrating sample magnetometer at room temperature. As shown in Figure 2, Fe_3_O_4_ presents the highest magnetization and α-Fe_2_O_3_ shows the lowest magnetization after heating at 500 °C. 

**Figure 3:**
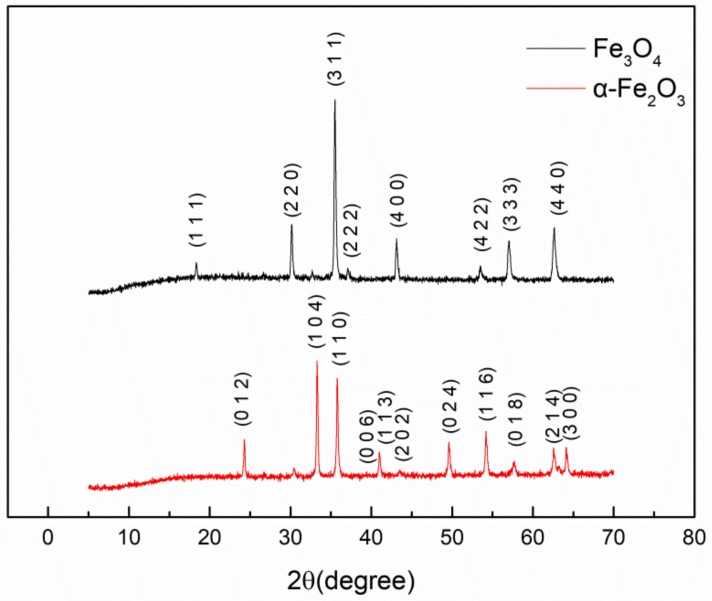
XRD patterns of Fe_3_O_4_ and α-Fe_2_O_3_. Particles were indexed according to the standard of ICDS Cards, 74-0748 and 79-0007 for Fe_3_O_4_ and α-Fe_2_O_3_, respectively.

As shown in Figure 3, the accordance between the peak positions of XRD patterns and ICDS cards of Fe_3_O_4_ and α-Fe_2_O_3_ particles were proved. And the XRD patterns are in coherence with ICDS cards of, pdf # 74-0748 (Fe_3_O_4_) and pdf # 79-0007 (α-Fe_2_O_3_). The results reveal that crystal form of Fe_3_O_4_ with heating is changed, while its own chemical composition is in accord with that of α-Fe_2_O_3_.

Moreover, the values of particle size are listed in Table 2. The particle size of α-Fe_2_O_3_ quite close to that of Fe_3_O_4_, which further indicates that the interaction between the two particles and matrix is similar.

### 3.3. Morphology

**Figure 4:**
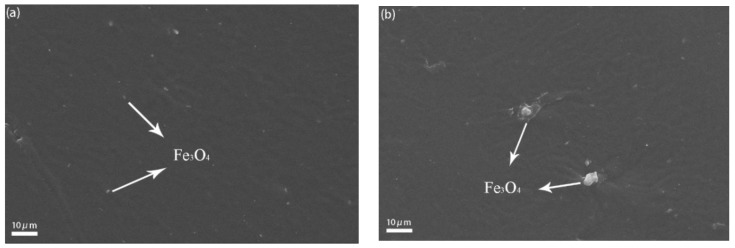

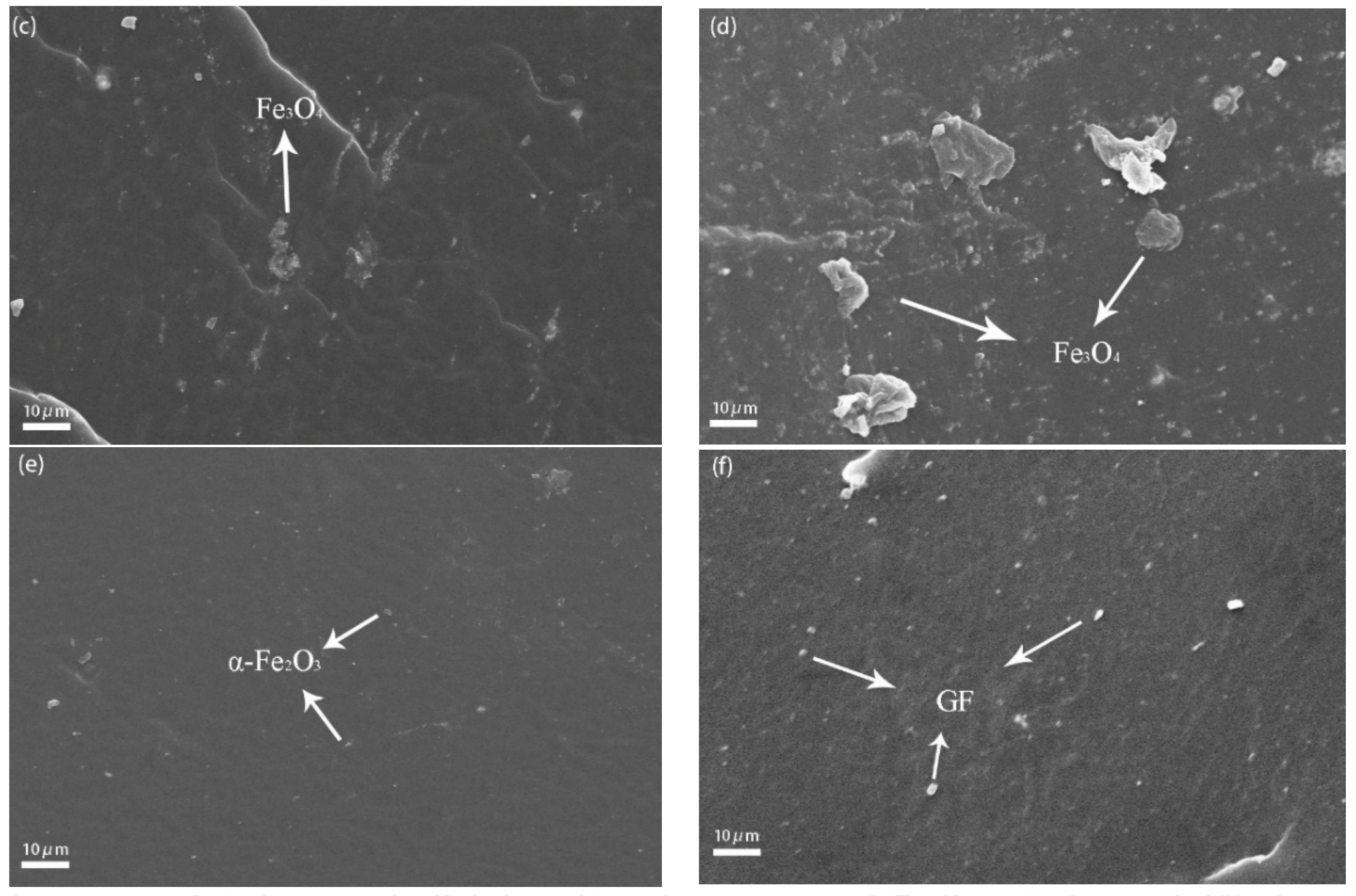
SEM images of (**a**) PBMA/0.5% Fe_3_O_4_ composites; (**b**) PBMA/1% Fe_3_O_4_ composites; (**c**) PBMA/2% Fe_3_O_4_ composites; (**d**) PBMA/5% Fe_3_O_4_ composites; (**e**) PBMA/1% α-Fe_2_O_3_ composites; (**f**) PBMA/1% GF composites.

The dispersion of the fillers was researched by SEM measurements on the brittle and snapped sample surface. As shown in Figure 4, in PBMA/Fe_3_O_4_ composites, the dispersion of Fe_3_O_4_ and their interfacial interactions with the PBMA matrix are critical for the damping properties of the composites. The dispersion of Fe_3_O_4_ in the PBMA matrix was homogeneous when 0.5% Fe_3_O_4_ was added. When the content of Fe_3_O_4_ was beyond 1%, the damping properties decreased slightly because of the aggregation of Fe_3_O_4_ (Figure 4c,d). α-Fe_2_O_3_ particles, after high temperature demagnetization, have good dispersibility in the PBMA matrix, as shown in Figure 4e. When the GF was added into the PBMA matrix, the dispersion of them was relatively homogeneous.

### 3.4. Damping Property of PBMA/Fe_3_O_4_ and PBMA/α-Fe_2_O_3_

Dynamic mechanical analysis (DMA) is widely used to determine material damping properties as functions of temperature, frequency, and stress [23] and to investigate the effect of an increasing content of inorganic particles on the damping properties of PBMA/Fe_3_O_4_ and PBMA/α-Fe_2_O_3_. Results of the DMA tests are presented in the form of loss factor, tan δ, as functions of temperature.

**Figure 5:**
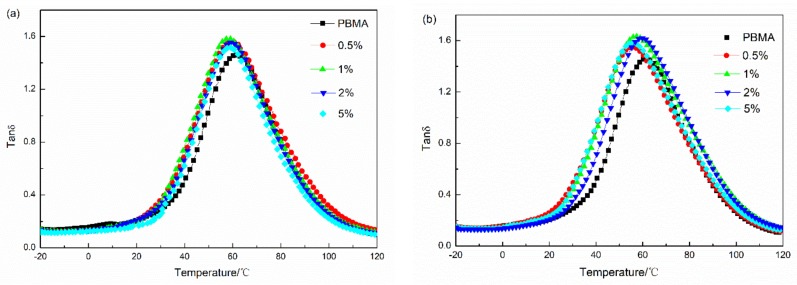
Temperature dependence spectra of tan δ of PBMA with different particles and different mass fraction at 1 Hz. (**a**) Fe_3_O_4_; (**b**) α-Fe_2_O_3_.

**Figure 6:**
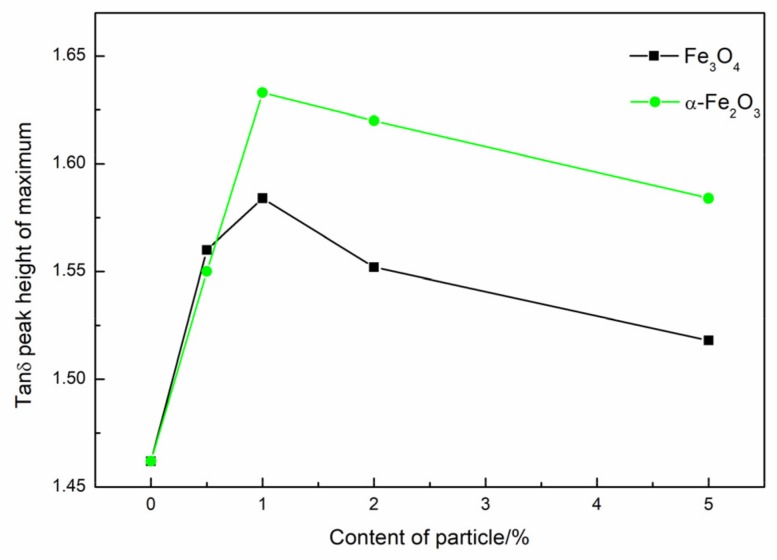
Effect of the content of inorganic particles on the peak height of PBMA blends.

Curves of the variations of tan δ with the temperature of Fe_3_O_4_ and α-Fe_2_O_3_ composites are shown in Figure 5 and Figure 6, respectively. The loss tangent (tan δ) is commonly called damping and is the evaluation measure of energy dissipation. The values of the maximum heights versus the loads of particles are shown by Figure 6. As is seen, Fe_3_O_4_ and α-Fe_2_O_3_ show similar influences on the maximum heights of tan δ. 

For Fe_3_O_4_, the maximum heights increased at first and then decreased; the largest points of maximum heights were located when the mass fraction of Fe_3_O_4_ was 1%. When Fe_3_O_4_ were added to the composites, internal friction between Fe_3_O_4_ and polymer chains, as well as friction between Fe_3_O_4_ particles, increased the rate of the dissipating energy. The damping properties were improved. The damping properties decreased slightly when the content of Fe_3_O_4_ was beyond 1% due to the aggregation of Fe_3_O_4_ decreasing the internal friction of the composites. Table 3 shows that all PBMA/Fe_3_O_4_ blends have efficient damping (tan δ > 0.3) over a wide temperature range of more than 64 °C. Moreover, the peak area under the tan δ temperature curves is abbreviated as TA, which is a measure of the energy dissipation of a transition process [24]. The TA values of PBMA/Fe_3_O_4_ composites are also summarized in Table 3. Compared with blank PBMA, PBMA/Fe_3_O_4_ composites exhibit relatively high TA values.

The glass transition temperature (T_g_) of the composites, taken at the maximum value of the tan δ, is determined by the DMA [25,26]. As observed from Figure 5a, increasing the mass fraction of Fe_3_O_4_ shifted the curve peak to low temperatures, with the incorporation of 1% Fe_3_O_4_ into the PBMA matrix leading to the greatest decrease in T_g_, referring to neat PBMA. This phenomenon can be interpreted as follows: With the inclusion of Fe_3_O_4_ into PBMA, the Fe_3_O_4_ particles interact with and shield carbanyl groups and, as a result, a lowered T_g_ was observed.

For α-Fe_2_O_3_, to improve the dispersion of Fe_3_O_4_ in the PBMA matrix in the present study, demagnetization of Fe_3_O_4_ (α-Fe_2_O_3_) was incorporated into the PBMA. The maximum tan δ of PBMA/α-Fe_2_O_3_ increased and the temperature range with tan δ > 0.3 became wider, as shown in Figure 5b and Table 3. The damping properties of the composites improved. Moreover, when the weight content of α-Fe_2_O_3_ was 1%, the tan δ reached 1.63, and the corresponding temperature range, with tan δ > 0.3, was about 103 °C. The results show the demagnetization of Fe_3_O_4_ (α-Fe_2_O_3_) could increase the internal friction due to homogeneous dispersion of α-Fe_2_O_3_ in the PBMA matrix (Figure 4e). Moreover, T_g_ of the α-Fe_2_O_3_ composites shifts to low temperature as seen from Figure 5b and it is more obvious to shift to low temperature comparing to Fe_3_O_4_ composites.

### 3.5. Damping Property of PBMA/GF

**Figure 7:**
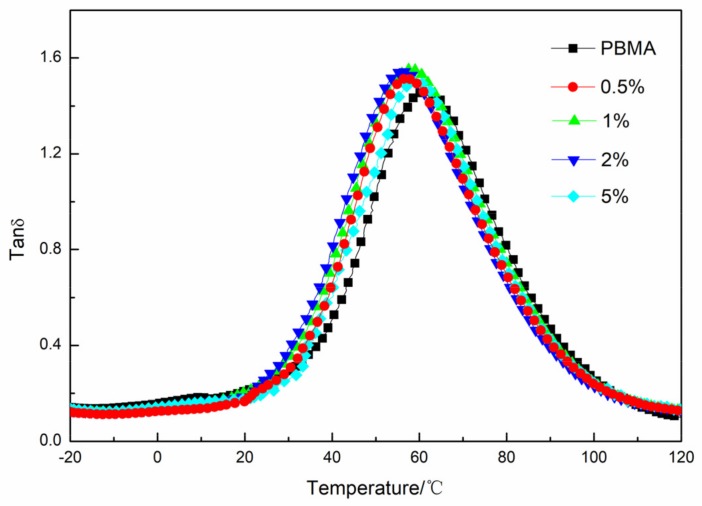
The temperature dependence of loss tangent (tan δ) at 1 Hz for PBMA and PBMA/GF.

**Figure 8:**
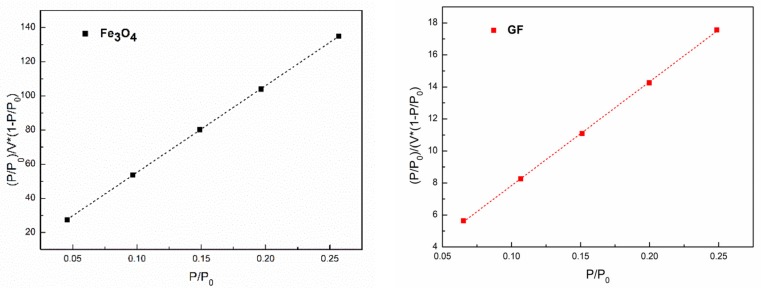
Brunauer-Emmet-Teller (BET) spectra of Fe_3_O_4_ and GF measured by a specific surface area and pore size tester. Fitting index, *R*^2^ = 0.99996, 0.99973, respectively.

As shown in Figure 7, the loss tangent, tan δ, of PBMA/GF composites increased compared with neat PBMA. However, Figure 7 and Table 4 demonstrated that the damping factor of PBMA/GF composites is lower than that of PBMA/Fe_3_O_4_ composites, although, Fe_3_O_4_ particles with a modified surface can better disperse in the PBMA matrix. These results are because the interfacial interaction in PBMA/GF hybrids is stronger than that in the PBMA/Fe_3_O_4_ hybrids, as shown in Table 3. The adhering of Fe_3_O_4_ to graphene, as shown in Figure 1, changes the interaction surfaces between particles and molecular chains that interact between Fe_3_O_4_ and PBMA and become the interaction between graphene and PBMA. GF particles have a larger specific surface area, as shown in Figure 8, which enhances interaction between GF and PBMA, thus, resulting in lower internal friction.

It is interesting that the glass transition temperature of PBMA/GF composites also shifts to low temperatures. The results are attributed to Fe_3_O_4_ adhering to graphene, which changes the interaction surfaces between particles and molecular chains as mentioned above. Moreover, graphene is a typical two-dimensional layered material. The layered structure is held together by van der Walls interactions, shown in Figure 1. An intercalation state or, even, exfoliation state of fillers is possibly formed due to the weak van der Walls interactions between adjacent layers. Furthermore, the layer of two-dimensional layered fillers could slip to some extent [27]. For example, Jiang et al. [28] prepared chlorinated butyl rubber/graphene oxide composites (CIIR/GO) and proposed a slippage of the lamellae to interpret the results that the T_g_ of CIIR/GO composites shifts towards low temperatures. The mechanism is also used to interpret our case in that the slippage of the lamellae could increase the mobility of PBMA chains so that the glass transition temperatures of PBMA/GF hybrids shift towards low temperatures.

### 3.6. Surface Properties of Inorganic Particles

To study the effects of the interaction between particles and PBMA on the damping property of composites, the results of a specific surface area and pore size tester were analyzed by the BET method; then, the surface areas of particles measured by BET (S_BET_) can be calculated by:

(1) $$\frac{P}{V\left(P_{0}-P\right)}=\frac{1}{V_{m}\times C}+\frac{C-1}{V_{m}\times C}\times \left(P/P_{0}\right)$$

(2) $$S_{\mathrm{BET}}=V_{m}\times A\times \sigma_{m}$$

where:

- V_m_ → single layer adsorption volume
- V → all adsorption volume
- P → partial pressure of adsorption thing
- P_0_ → saturated vapor pressure of adsorption thing
- C → BET constant
- A → Avogodro constant ($6.023\;\times 10^{23}$/mol)
- $\sigma_{m}$ → the sectional area of an adsorption thing (for the adsorption of $N^{2}$, $\sigma_{m}$ = $16.2\;\times 10^{-20}\;m^{2})$.

**Figure 9:**
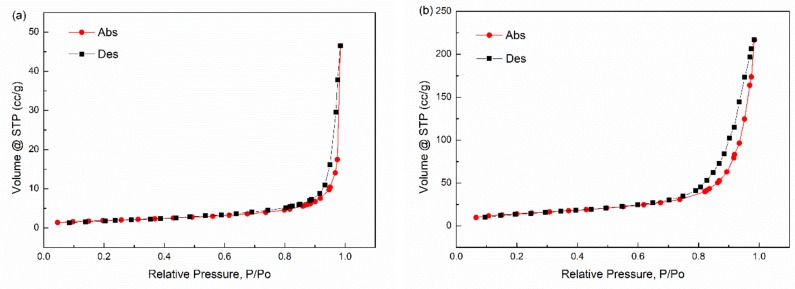
Spectra of nitrogen absorption and desorption trends for BET. (**a**) Fe_3_O_4_; (**b**) GF.

Thus, the $\left(P/P_{0})/V\times (1-P/P_{0}\right)$ vs. ($P/P_{0}$) should be linearity. The linear fitting was used and the results (BET spectra) are plotted in Figure 8. According to the slope and intercept of these fitting lines, we can obtain the value of V_m_. In Figure 8, all dots can be linearly fitted in some degree (*R*^2^ > 0.9). The values of the surface areas measured by BET (S_BET_) are listed in Table 5. According to Table 5, the S_BET_ of GF is larger than that of Fe_3_O_4_, which may be caused by graphene, which has a larger specific surface. Moreover, to better understand the surface distribution of the materials, the spectra of nitrogen absorption and desorption for BET is shown in Figure 9.

## 4. Conclusions

In this study, hybrid composites based on poly(butyl-methacrylate), Ferriferous oxide(Fe_3_O_4_), Graphene/Fe_3_O_4_(GF), and demagnetization of Fe_3_O_4_ (α-Fe_2_O_3_) were successfully prepared. The SEM and EDS results show that Graphene/Fe_3_O_4_ (GF) was synthesized and that α-Fe_2_O_3_ can be finely dispersed in the PBMA matrix. VSM results revealed that α-Fe_2_O_3_ showed the lowest magnetization after heating at 500 °C. The SEM results show that dispersion of α-Fe_2_O_3_ in the PBMA matrix is better than that of Fe_3_O_4_ and the BET results, exhibiting interfacial interactions between PBMA and GF, is stronger than that between PBMA and Fe_3_O_4_. The DMA results indicate that the damping properties of PBMA can be improved by filling Fe_3_O_4_, GF and α-Fe_2_O_3_. When the weight content of α-Fe_2_O_3_ was 1%, the PBMA/α-Fe_2_O_3_ had the best damping performance, with the tan δ reaching 1.63 and the corresponding temperature range, with tan δ > 0.3, being about 103 °C. Moreover, it is also worth mentioning that the T_g_ of the composites shifted to low temperatures. This result needs to be further studied.

## Figures and Tables

**Table 1:** Formulations of poly (butyl-methacrylate) inorganic particles composites.

Material	Weight (g)
PBMA	19.9/19.8/19.6/19.0
Fe_3_O_4_	0.1/0.2/0.4/1.0
α-Fe_2_O_3_	0.1/0.2/0.4/1.0
GF	0.1/0.2/0.4/1.0

**Table 2:** Value of particle size.

Sample	d (0.5)
Fe_3_O_4_	2.202 μm
α-Fe_2_O_3_	2.110 μm

**Table 3:** The damping properties of PBMA/Fe_3_O_4_ and PBMA/α-Fe_2_O_3_ blends.

Sample Code	Tan δ Max		Temperature Range of Tan δ > 0.3			TA (Tan δ > 1.0)
**PBMA/Fe_3_O_4_**	**Value**	**T/°C**	**T_1_/°C**	**T_2_/°C**	**ΔT/°C**	
0%	1.46	61.19	30.80	98.00	67.20	7.39
0.5%	1.56	59.66	26.87	100.83	73.96	10.59
1%	1.58	57.72	28.30	97.97	69.67	10.58
2%	1.55	59.06	28.43	97.02	68.59	9.21
5%	1.52	58.63	30.84	94.86	64.02	8.08
**PBMA/α-Fe_2_O_3_**						
0%	1.46	61.19	30.80	98.00	67.20	7.39
0.5%	1.55	55.3	22.79	98.34	75.55	10.59
1%	1.63	56.81	25.19	103.00	77.81	13.43
2%	1.62	59.05	27.83	102.51	74.68	12.71
5%	1.58	55.58	25.16	99.80	74.64	11.74

**Table 4:** The damping properties of PBMA/GF.

Sample Code	Tanδ Max		Temperature Range of Tanδ > 0.3			TA (Tanδ > 1.0)
PBMA/GF	Value	T/°C	T_1_/°C	T_2_/°C	ΔT/°C	
0.5%	1.52	56.88	30.13	95.68	65.55	8.11
1%	1.55	57.63	27.95	96.36	68.41	9.78
2%	1.54	55.95	27.08	94.68	67.60	9.27
5%	1.50	58.72	33.74	96.12	62.38	7.80

**Table 5:** Values of S_BET_.

Samples	S_BET_
Fe_3_O_4_	6.808
GF	52.565
